# Medical Association of Central New York

**Published:** 1900-07

**Authors:** 


### MEDICAL ASSOCIATION OF CENTRAL NEW YORK.

The following discussion was had upon Dr. Wm. P. Spratling's paper, entitled, The Treatment of Epilepsy, which was read before the Medical Association of Central New York, at Syracuse, October 17, 1899, and which appeared in the June number of the JOURNAL:

DISCUSSION.

Dr. WM. C. KRAUSS, Erie—I wish to thank Dr. Spratling personally for the pleasure and profit which I have derived from listening to his paper; and I think I voice the sentiment of the society when I thank him on behalf of the society on the same grounds.

To me, this paper is as important and interesting a one as I have heard in a long, long time. It is important from two points of view: In the first place, it deals with treatment; and in the second place, it deals with a disease as difficult and as hard to which to apply the proper treatment as any with which physicians have to contend. I think, in all our medical societies, we have too few papers on the treatment of diseases. We have too many on pathology and on theories and perhaps diagnosis, but we have too few on the treatment of disease: and that virtually is what we come together for, to learn something about treating and caring for the sick.

Then, another point, the treatment of epilepsy has been changing, and I believe the physical treatment, the treatment of making a strong mind by making the arm or the leg strong, is an important point which the doctor has so nicely brought out.

I notice the doctor did not say anything about the surgical treatment of epilepsy. Four or five or six years ago, we heard nothing but the surgical treatment of epilepsy, and all our cases had to be sent to the surgeon for an operation. Now we look upon the surgical treatment of epilepsy as a sort of a fallacy, a fad. Of all the cases that I have had operated on, I think there is not a single one where the operation has had any permanent benefit. For the time being, for perhaps a few months, the patient would be benefited but after that, would drift into the same habit of having their attacks, and they would come on with more force and frequency until they became confirmed chronic epileptics.

I think the point which the doctor brought out about the habit of getting an attack, the habit which these patients fall into so easily, the habit which the mind has of doing a certain thing a certain number of times and always doing it in a certain way and along certain lines, is one of the most important things which the doctor brought up in this paper. It behooves us all, in these cases of epilepsy, to make a careful and thorough examination, not only once, but repeatedly; and, then, if there is any little symptom regarding the organs of sense, send those patients to our eye friends and to our ear friends and to our nose friends, and have all the reflex phenomena pointed out to us and removed before we go on with the regular course of bromide treatment.

The cases of psychical epilepsy and those cases in which insanity appears in epilepsy, I think are also important. I remember a case of psychical epilepsy that occurred in the vicinity of Buffalo about eight years ago, where a young girl in one of these psychical conditions, killed one little girl and tried to kill another. The case was tried in court and the condition of psychical epilepsy was pointed out to the court, and the jury very wisely exonerated the girl from punishment. And yet, a few days afterwards, the judge who sat on the bench at that time, told Dr. Andrews that in his opinion the girl was shamming and it was a case of justice gone wrong. It was not. It was a case of justice gone right. I think these cases of epilepsy that show any temper and that have any spells of discontentment, are the most dangerous cases we have to deal with, because at any time homicidal tendencies creep out, and before we know it, we have a serious condition of affairs on our hands. All such cases should be taken from home just as soon as possible and put under proper restraint.

During the past summer, I had occasion to learn something about the way the Germans treat diseases, and I found that the Flechsig treatment had been virtually abandoned in northern Germany. The Germans have a queer way of treating their patients. When they have a hard case, instead of working at it in the way Americans do, they simply send them to some bath, some watering-place, to Carlsbad or Baden-Baden, and the chronic cases are off their minds and, of course, they get the credit of doing something very bright.

In conclusion, I wish to move a vote of thanks to Dr. Spratling for the very interesting address which he has given us.

Seconded. Carried.

Dr. E. B. ANGELL, Monroe—I wish to emphasise just one point that Dr. Spratling very wisely dwelt upon, but I believe it will be for your good if you hear it repeated, and that is the way you have to deal with a case of epilepsy. Don't diagnosticate it simply as epilepsy. That has been the fault of the general practitioner time and time again, especially in dealing with that phase of nervous disease.

If you haven't much time to give to a patient when he first comes to your office, spend the time in studying the case. Give him some bromide or anything you please. When he comes back again, study the case. It is just as it is in the building of roads. A good engineer will tell you the first thing to do is drainage, the next thing to do is drainage and the third thing is drainage. It is all drainage. And so it is with the diagnosis and treatment of epilepsy. The important thing is studying the case. It is always studying the case because, after all, the essential feature of epilepsy, so far as we know it now at the present time, is the establishment of the epileptic habit, the expression of the attack; and what we want to do is to find out the way in which that attack is developed, find out what is wrong with this brain by which that attack is made possible and just so far as possible correct those conditions.

Very little is said today about the bromide treament, about the medicines used in masking the disease and suspending the attacks. The important matter is to recognise that you are dealing with a habit, and just so far as possible, you want to teach the individual to control it. Therefore, study your case.

Dr. H. B. HAWLEY, Onondaga—I don't want to say anything about epilepsy, but the remark of Dr. Krauss brought a thought to my mind which I deem it advisable to mention, and that is the remark that the jury found this patient guilty but the judge found her in his mind not guilty. It seems to me that a great many of these cases which are dependent upon the judgment of some juror or some judge as to the question of guilt are dependent upon the wrong person, and I would like to say a word right here to this representative body of men in the line that will bring about, perhaps, at some future day, a righting of this wrong and of the subject that has been spoken of. That is to say, the proper handling, the proper committee or the proper people to pass upon these questions of sanity or insanity, of responsibility or irresponsibility for overt acts against law that are done by incompetent people. It brings to my mind the case of the justice here in Syracuse who made a remark on the bench that he had sat on the bench with a temperature of 103 or 4 and he was competent to judge of cases and therefore that an individual who was suffering from the same temperature was competent to sit in the witness stand or in the dock the same as though he were well.

